# Identification of *DHX40* as a candidate susceptibility gene for colorectal and hematological neoplasia

**DOI:** 10.1038/s41375-023-02021-9

**Published:** 2023-09-11

**Authors:** Alisa Olkinuora, Taina T. Nieminen, Suvi Douglas, Anni Kauppinen, Mika Kontro, Juho Väänänen, Matti Kankainen, Ari Ristimäki, Markus Mäkinen, Päivi Lahermo, Caroline Heckman, Janna Saarela, Milla Salonen, Anna Lepistö, Heikki Järvinen, Jukka-Pekka Mecklin, Outi Kilpivaara, Ulla Wartiovaara-Kautto, Kimmo Porkka, Päivi Peltomäki

**Affiliations:** 1 Department of Medical and Clinical Genetics, University of Helsinki, 00014 Helsinki, Finland; 2 Applied Tumor Genomics Research Program, Faculty of Medicine, University of Helsinki, 00014 Helsinki, Finland; 3 Department of Hematology, Helsinki University Hospital, Comprehensive Cancer Center and University of Helsinki, 00014 Helsinki, Finland; 4 HiLIFE Institute for Molecular Medicine Finland (FIMM), University of Helsinki, 00014 Helsinki, Finland; 5 Foundation for the Finnish Cancer Institute, 00014 Helsinki, Finland; 6 HUSLAB Laboratory of Genetics, HUS Diagnostic Center, HUS, Helsinki University Hospital, 00029 Helsinki, Finland; 7 Hematology Research Unit Helsinki, University of Helsinki, 00014 Helsinki, Finland; 8 iCAN Digital Precision Cancer Medicine Flagship, Helsinki, Finland; 9 Translational Immunology Research Program and Department of Clinical Chemistry and Hematology, University of Helsinki, 00014 Helsinki, Finland; 10 Department of Pathology, HUSLAB, HUS Diagnostic Center, Helsinki University Hospital and University of Helsinki, 00014 Helsinki, Finland; 11 Research Unit of Cancer and Translational Medicine, Department of Pathology, 90014, University of Oulu, and Department of Pathology, Oulu University Hospital, OYS, 90029 Oulu, Finland; 12 Centre for Molecular Medicine Norway, NCMM, University of Oslo, 0318 Oslo, Norway; 13 Department of Veterinary Biosciences, University of Helsinki, 00014 Helsinki, Finland; 14 Folkhälsan Research Center, 00290 Helsinki, Finland; 15 Department of Abdominal Surgery, Helsinki University Hospital and University of Helsinki, 00014 Helsinki, Finland; 16 Department of Education & Research and Surgery, Jyväskylä Central Hospital, 40620 Jyväskylä, Finland; 17 Department of Sports & Health Sciences, Jyväskylä University, 40014 Jyväskylä, Finland

**Keywords:** Cancer genetics, Haematological cancer

## To the Editor:

Accumulating evidence suggests shared susceptibility factors between colorectal and hematological malignancies, in line with observations of cross-cancer associations of multiple cancer predisposing genes [1]. Familial colorectal cancer type X (FCCTX) refers to colorectal cancer families that fulfill the diagnostic (Amsterdam) criteria for Lynch syndrome but lack DNA mismatch repair (MMR) defects. The underlying genes are mostly unknown. We recently linked inactivation of *RPS20* (ribosomal protein S20) to FCCTX predisposition [2]. The phenotypic spectrum of *RPS20* was later expanded to include Diamond Blackfan anemia [3] thus connecting colorectal and hematological carcinogenesis. Observations of inherited variants in *DDX41*, a DEAD box RNA helicase gene, causing susceptibility to myelodysplastic syndrome and myeloid leukemias, offer additional proof of the important regulatory roles of ribosome biosynthesis and RNA processing in cancer [4]. *DDX41* variants are currently known to represent the most common germline alterations in adult myelodysplastic syndromes, accounting for 0.5–4% of all acute myeloid leukemia or myelodysplastic syndrome cases in adults [5].

**Fig. 1:**
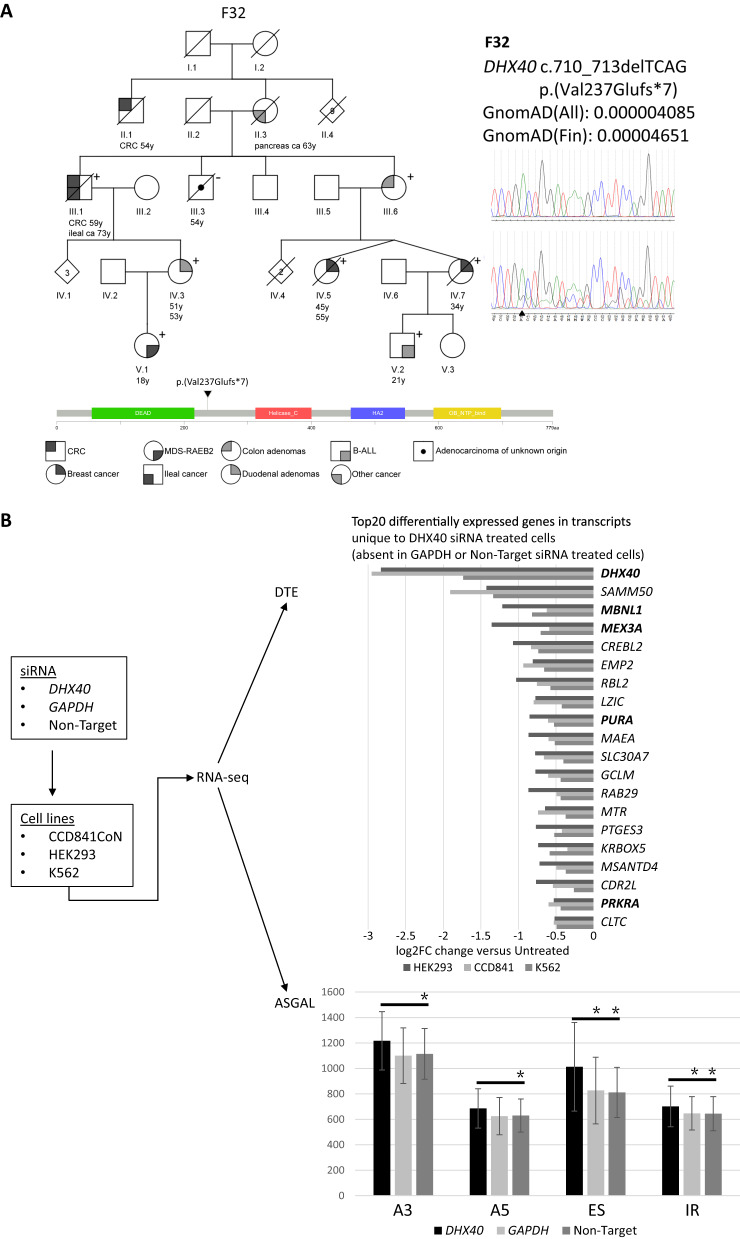
Genetic and functional characterization of the *DHX40* c.710_713delTCAG variant. **A** Pedigree of F32. Numbers below the symbols are patient identifiers. Arrow denotes the index person. Tumor manifestations and age at diagnosis (years) are given below patient symbols. Non-essential pedigree features have been excluded or modified to protect confidentiality. A plus sign (+) denotes the presence of the variant in a heterozygous state and a minus sign (-) the absence of the variant. Sequence chromatogram on the right displays the normal (top) and altered sequence (bottom), where arrowhead denotes the site of the germline change. Lollipop diagram (bottom) indicates location of the predisposing variant against the main functional domains of the encoded protein. The functional domains of DHX40 are: DEAD, DEAD/H box helicase domain; Helicase_C, Helicase conserved C-terminal domain; HA2, Helicase associated domain; OB_NTP bind, Oligonucleotide/oligosaccharide-binding (OB)-fold. **B** Consequences of siRNA-mediated knockdown of *DHX40*. Workflow of siRNA experiments on CCD841CoN, HEK293, and K562 cell lines is shown on the left. Results from analyses of RNA-sequencing data for differential transcript expression (DTE) and novel splicing events (by the ASGAL tool) (see Supplementary Materials and Methods) are depicted on the right. The bar graph of DTE analysis displays top 20 differentially expressed genes among 71 unique transcripts shared between the three cell lines. Genes whose products participate in RNA metabolism are **in bold** (see Supplementary Fig. 3 for all 71 genes). The ASGAL analysis shows the number of novel splicing events detected after treatment with *DHX40*-siRNA, *GAPDH*-siRNA, or non-target siRNA, vs. untreated cells, and stratified by the type of splicing alteration (A3, alternative 3’ site; A5, alternative 5’ site; ES, exon skipping; IR, intron retention). Splicing events for all three cell lines (HEK293, K562, and CCD841CoN) were combined. Asterisk denotes statistically significant differences (*p* < 0.0001 for ES and *p* < 0.05 for all other events, by pairwise chi-square test with FDR correction) in the number of specific types of splice events after *DHX40*-siRNA treatment vs. *GAPDH*-siRNA or non-target siRNA treatment.

We conducted exome sequencing (ES) on 28 unexplained FCCTX families to identify their predisposing genes (Supplementary Materials and Methods, Supplementary Table 1). The investigations described in this paper were approved by the Institutional Review Boards of the Helsinki University Hospital, Helsinki, Finland (approval nos. 466-46-2001, 206/13/03/03/2016 and 303/13/03/01/2011). Family F32 with myelodysplastic syndrome/acute leukemia coexisting with colorectal cancer caught our attention (Fig. 1A). All variants fulfilling our selection criteria (high quality, minor allele frequency <0.001, nonsynonymous, and predicted pathogenic with at least 5/6 in silico programs if missense) are listed for the index individual III.1 in Supplementary Table 2. His granddaughter V.1 presented with an unusual form of hematologic disorder at the age of 18 years. Initially, the investigations started due to pancytopenia which was treated with allo-hematopoietic stem cell transplantation (HSCT). The disease relapsed 2 years later with blood counts: hemoglobin 9.5, platelets 50, neutrophils 0.7. At that time, morphological examination of the bone marrow showed dysplasia in megakaryopoiesis and excess of blasts, and was subsequently diagnosed as myelodysplastic syndrome (MDS)/refractory anemia with excess blasts, type 2 (RAEB2) [6]. When variant sharing in the key affected members III.1, IV.3, and V.1 was set as a requirement, the only variant that remained was a heterozygous truncating variant affecting the DEAH-box RNA helicase gene *DHX40* (NM_024612.4:c.710_713delTCAG, p.Val237GlufsX7) (Fig. 1A). The variant is predicted to lead to the deletion of the helicase domain and all downstream C-terminal portion of DHX40. Seven of eight cancer-affected members had this variant (the only one without was individual III.3 with adenocarcinoma of unknown origin).

To functionally characterize the *DHX40* variant, we evaluated its expressional consequences on RNA and protein level, undertook tumor studies to analyze somatic variants, changes in gene methylation and DHX40 protein expression, and conducted siRNA analyses of cell lines (see Supplementary Materials and Methods). By a primer extension assay, the dosage ratios of mutant to wild-type alleles were reduced up to fivefold (Supplementary Fig 1A), likely reflecting nonsense-mediated RNA decay. In agreement, Western blot analysis with N-terminal DHX40 antibody showed no visible truncated protein (Supplementary Fig. 2A). However, a truncated product of expected size could be clearly visualized by transfecting HEK293 cells with eGFP-tagged DHX40 expression constructs (Supplementary Fig. 2B). To evaluate if Knudson’s two-hit mechanism applied to *DHX40*, available neoplastic tissues from individuals heterozygous for the *DHX40* c.710_713delTCAG variant were examined for somatic variants, loss of the wild-type allele, and promoter methylation of *DHX40*. There was no unequivocal evidence of “second hits” (Supplementary Fig. 1B, Supplementary Table 3, Supplementary Table 4), supporting the idea that loss of function of the constitutionally mutant allele alone was sufficient for tumorigenesis, without a second hit (haploinsufficiency).

As no suitable RNA samples from the patients were available for investigations of the consequences of *DHX40* inactivation on global transcriptomes, we undertook siRNA experiments on cell lines representing normal (CCD841) or cancer tissues (HEK293 and K562) of different cellular origins (Fig. 1B, Supplementary Materials and Methods), followed by RNA-sequencing. We observed 71 differentially expressed transcripts (*q*-value < 0.01) unique to *DHX40*-siRNA-treated cells and shared by all three cell lines (Fig. 1B, Supplementary Fig. 3). Genes functioning in RNA metabolism (RNA helicase, RNA binding, or transcription-related function) were enriched, comprising 31% of the unique transcripts. ASGAL (Alternative Splicing Graph ALigner) directly aligns RNA-seq data to a splicing graph, which results in a list of novel splice events in respect to gene annotation. By ASGAL analysis, *DHX40*-siRNA-treated cells exhibited a 13% increase in novel splice events, compared to *GAPDH*-siRNA-treated or non-target siRNA-treated cells (Fig. 1B). *DHX40*-siRNA treatment associated with elevation of all types of splice events, especially exon skipping.

No germline variants suspected pathogenic were detectable in *DHX40* or other DEAD/H box genes (from here on, referred to as DDX/DHX genes) in the remaining 27 FCCTX families. However, one family displayed a heterozygous truncating variant in a related RNA helicase gene, *TDRD9* (NM_153046.2:c.2261delC, p.Thr754IlefsX11) that co-segregated with colorectal cancer in two siblings. This finding further strengthens the importance of impaired RNA metabolism behind hereditary cancer susceptibility.

**Fig. 2:**
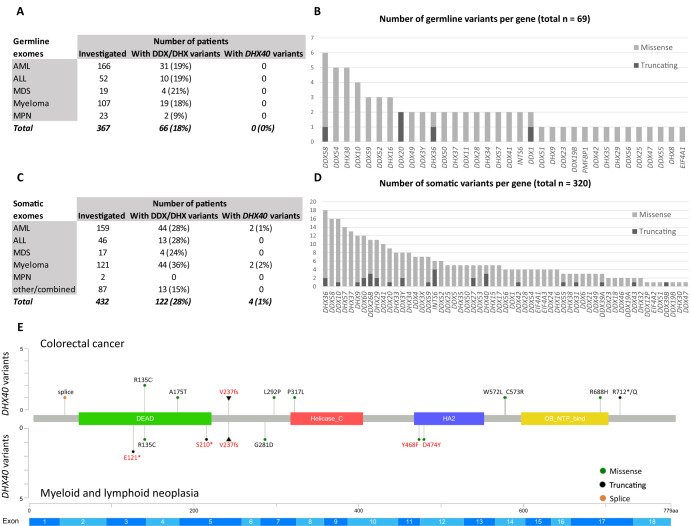
Prevalence of DDX/DHX variants in hematological neoplasia and summary of *DHX40* variants in colorectal and hematological neoplasia. **A**, **B** Germline exomes (fibroblasts) from our hospital-based hematological series. Variant selection criteria were the same as for FCCTX, except that in silico evaluations were based on five programs instead of six. Bar chart in 3B includes DDX/DHX genes affected by at least one variant. See Supplementary Table 5 for variant details. **C,** **D** Somatic exomes (leukemic bone marrow) from our hospital-based hematological series. Nonsynonymous variants with allele frequency (VAF) 1% or higher and somatic *p* value < 0.01 by VarScan2 were selected. The “other/combined” group in 3C consists of mainly combined diagnoses of different hematological lineages. The four cases of somatic *DHX40* variants listed in 3C include p.S210*, VAF 7% (AML_3 with recurrent AML); p.Y468F, VAF 7% (AML_43 with polycythemia vera and subsequent acute promyelocytic leukemia); p.D474Y, VAF 7% (MM_14 with multiple myeloma); and p.E121*, VAF 37% (MM_20 with recurrent multiple myeloma). Bar chart in 3D includes DDX/DHX genes affected by at least one variant. See Supplementary Table 6 for variant details. **E** Lollipop diagram of germline (triangle) and somatic (circle) variants in *DHX40* present in colorectal, myeloid, and lymphoid neoplasia. Variants shown in red font are reported in this article, and variants in black are from publicly available datasets in cBioPortal (see Supplementary Materials and Methods). The germline variant p.V237Efs comprised both colorectal and myeloid/lymphoid phenotypes (Fig. 1A). See legend for Fig. 1A for functional domains of DHX40 (the corresponding exons of *DHX40* are underneath the diagram).

Next, we ascertained close to 400 patients with acute leukemia, myelodysplastic syndrome, or myeloma with germline and/or somatic exomic data available from a hospital-based repository and analyzed their samples for possibly pathogenic variants in DDX/DHX genes. Sixty-six skin fibroblast samples out of 367 (18%) revealed a possibly pathogenic germline variant in at least one DDX/DHX gene (Fig. 2A). Most variants were of the missense type (Fig. 2B). According to the FinnGen database (finngen.fi), of single nucleotide variants included in their genome-wide association study and located nearby our most frequently affected genes, *DDX58*, *DDX54*, *DHX38*, and *DDX10* showed significant (*p* < 10^−4^) association to several hematological malignancies. Additionally, *DDX10* showed significant (*p* < 10^−4^) association to colon adenocarcinomas, and *DDX54* to benign colon tumors. While *DHX40* was not affected with any such germline alterations that would fulfill our stringent selection criteria, the common p.M1I pathogenic loss of-function variant of *DDX41* [5] was detected twice. One hundred and twenty-two neoplastic bone marrow samples out of 432 (28%) revealed at least one possibly pathogenic somatic DHX/DDX single-nucleotide variant or small indel variant (mostly of the missense type), including *DHX40* variants in four (two with AML and two with myeloma) (Fig. 2C, D). The nonsense variant NM_024612.4:c.361G > T, p.E121* was particularly noteworthy being present in two consecutive specimens of CD138+ cells taken with a 15-month interval from a patient with multiple myeloma, and having a high variant allele frequency (37%) in both samples. This variant (together with p.S210* identified in an AML patient) affects the DEAD/H box helicase domain and is predicted to give rise to a severely truncated protein (Fig. 2E).

In summary, we describe a rare truncating germline variant of *DHX40* in a multi-generation family with hematological and solid malignancies. Loss-of-function nature of the variant, co-segregation with neoplasia phenotypes, and functional evidence suggest a role in cancer predisposition. Our findings add *DHX40* as a new candidate to the growing list of RNA metabolism-related genes that may underlie predisposition to FCCTX [2, 7] and myeloid disorders [4]. Moreover, our findings from a large hospital-based patient series suggest the involvement of the broader DDX/DHX gene family in hematological malignancies.

Our *DHX40* findings resemble those earlier described for *DDX41* in several respects. First, while *DDX41* primarily associates with hematological neoplasia, solid malignancies, especially prostate cancer, colorectal cancer, and melanoma, have been reported in individuals with pathogenic germline variants of *DDX41* [8]. Second, our patient V.1 from F32 responded well to combination therapy (clofarabin, plerixafor and lenalidomide) and had no need for additional therapy regimens after a second allo-HSCT following the combination therapy; the patient has remained in remission for over 10 years. Interestingly, sensitivity of *DDX41*-mutated patients to lenalidomide treatment has been described in the literature [4]. Third, although the frameshift nature of the *DHX40* germline variant implied loss of function, no apparent “second hits” were detectable in neoplastic tissues from individuals from F32 (Supplementary Table 3), a situation compatible with dominant-negative mechanism or alternatively, haploinsufficiency. Experience from *DDX41* suggests haploinsufficiency: although individuals with pathogenic germline variants often acquire somatic *DDX41* variants as second hits, the latter occur at low (0–20%) frequencies in bone marrow cells because biallelic alterations are not compatible with proliferating hematopoietic cells [9, 10]. It is likewise possible that biallelic *DHX40* defects are not tolerated. Interestingly, a duodenum adenoma from F32 showed a mosaic loss of DHX40 protein by immunohistochemical analysis (Supplementary Fig. 1C), suggesting that biallelic defects could be present, but in only a small proportion of tumor cells. Fourth, *DDX41*-deficient blast cells were shown to exhibit aberrant exon skipping and retention [4] resembling our findings from siRNA-mediated knockdown of *DHX40* in cell lines (Fig. 1B).

At least 59 established DDX/DHX helicases are known [11]. In our hospital-based hematological series, DDX/DHX genes were fairly frequently affected by missense variants and occasionally (7–9%) by truncating variants (Fig. 2A–D) Although several DDX/DHX genes pinpointed in this series showed significant association to hematological and colorectal neoplasia according to public databases, the true significance of the finding remains to be determined by additional studies.

Apart from ATP-dependent RNA-duplex unwinding, DDX/DHX proteins participate in the regulation of long and short non-coding RNAs, pre-mRNA splicing, ribosome biogenesis, and many other cellular functions [11–14]. Besides shedding light to the basic biological mechanisms of neoplasia, our results are clinically relevant since DDX/DHX variant status may guide therapy options ([4] and this study); moreover, participation in translational control makes DDX/DHX helicases attractive targets for novel anti-cancer therapies [15]. Our findings encourage additional investigations on *DHX40* as well as studies into other DHX/DDX genes as possible predisposing factors for colorectal and hematological neoplasia.

### Supplementary information

Supplementary Materials and Methods

Legends for Supplementary Figures

Supplementary Figure 1

Supplementary Figure 2

Supplementary Figure 3

Supplementary Table 1

Supplementary Table 2

Supplementary Table 3

Supplementary Table 4

Supplementary Table 5

Supplementary Table 6

## Data Availability

Raw RNA-seq data and DTE analysis results from siRNA treated cell lines can be obtained from GSE228991. Our IRB approvals do not allow sharing of raw sequencing data from patients. All variants fulfilling our filtering criteria can be found in the Supplementary Files. Requests to access additional datasets should be directed to the corresponding authors.

## References

[1] Huang KL, Mashl RJ, Wu Y, Ritter DI, Wang J, Oh C (2018). Pathogenic germline variants in 10,389 adult cancers. Cell.

[2] Nieminen TT, O’Donohue M-F, Wu Y, Lohi H, Scherer SW, Paterson AD (2014). Germline mutation of rps20, encoding a ribosomal protein, causes predisposition to hereditary nonpolyposis colorectal carcinoma without dna mismatch repair deficiency. Gastroenterology.

[3] Bhar S, Zhou F, Reineke LC, Morris DK, Khincha PP, Giri N (2020). Expansion of germline RPS20 mutation phenotype to include Diamond-Blackfan anemia. Hum Mutat.

[4] Polprasert C, Schulze I, Sekeres MA, Makishima H, Przychodzen B, Hosono N (2015). Inherited and somatic defects in DDX41 in myeloid neoplasms. Cancer Cell.

[5] Quesada AE, Routbort MJ, DiNardo CD, Bueso-Ramos CE, Kanagal-Shamanna R, Khoury JD (2019). DDX41 mutations in myeloid neoplasms are associated with male gender, TP53 mutations and high-risk disease. Am J Hematol.

[6] Hong M, He G (2017). The 2016 revision to the world health organization classification of myelodysplastic syndromes. J Transl Int Med.

[7] Bellido F, Sowada N, Mur P, Lázaro C, Pons T, Valdés-Mas R (2018). Association between germline mutations in BRF1, a subunit of the RNA polymerase III transcription complex, and hereditary colorectal cancer. Gastroenterology.

[8] Churpek JE, Smith-Simmer K. DDX41-associated familial myelodysplastic syndrome and acute myeloid leukemia. In: Margaret P Adam, Ghayda M Mirzaa, Roberta A Pagon, Stephanie E Wallace, Lora JH Bean, Karen W Gripp, Anne Amemiya, editors. GeneReviews*.* University of Washington: Seattle; 2021.

[9] Chlon TM, Stepanchick E, Hershberger CE, Daniels NJ, Hueneman KM, Kuenzi Davis A (2021). Germline DDX41 mutations cause ineffective hematopoiesis and myelodysplasia. Cell Stem Cell.

[10] Makishima H, Saiki R, Nannya Y, Korotev S, Gurnari C, Takeda J (2023). Germ line DDX41 mutations define a unique subtype of myeloid neoplasms. Blood.

[11] Cai W, Xiong Chen Z, Rane G, Satendra Singh S, Choo Z, Wang C, et al. Wanted DEAD/H or alive: helicases winding up in cancers. *J Natl Cancer Inst.* 2017;109:djw278. 10.1093/jnci/djw278 28122908

[12] Rocak S, Linder P (2004). DEAD-box proteins: the driving forces behind RNA metabolism. Nat Rev Mol Cell Biol.

[13] Liu YC, Cheng SC (2015). Functional roles of DExD/H-box RNA helicases in Pre-mRNA splicing. J Biomed Sci.

[14] Russon MP, Westerhouse KM, Tran EJ (2021). Transcription, translation, and DNA repair: new insights from emerging noncanonical substrates of RNA helicases. Biol Chem.

[15] Heerma van Voss MR, van Diest PJ, Raman V (2017). Targeting RNA helicases in cancer: the translation trap. Biochim Biophys Acta Rev Cancer.

