# Supplementary material for: Identification of DHX40 as a candidate susceptibility gene for colorectal and hematological neoplasia
Source: Leukemia. 2023 Sep 11;37(11):2301–5. doi: 10.1038/s41375-023-02021-9 (PMC10624609; doi:10.1038/s41375-023-02021-9)
Supplement: Supplementary file 1 — Supplementary Materials and Methods [file 41375_2023_2021_MOESM1_ESM.docx]

SUPPLEMENTARY MATERIALS AND METHODS (Olkinuora et al.)

**Patients and samples**

Familial colorectal cancer type X (FCCTX)

Twenty-eight FCCTX families from Finland, with no MMR defects in tumor tissue or germline [1], were investigated. Twenty families fulfilled the strict Amsterdam (AI or AII) criteria, without the age criterion [2, 3] whereas 8 families fulfilled the revised Bethesda guidelines [4]. The number of patients diagnosed with colorectal cancer varied from 2 to 7 per family, and the mean age at onset of colorectal cancer was 59.6 years. FCCTX families were recruited through the nationwide Hereditary Colorectal Cancer Registry of Finland or the Helsinki University Hospital and the Jyväskylä Central Hospital. Blood, EBV-transformed lymphoblasts, and tumor tissues were used for nucleic acid extraction from index cases and available family members.

Case V.1 from family 32

At the age of 18 years, case V.1 from family 32 (Fig. 1A) presented with an unusual form of hematologic disorder, subsequently diagnosed as myelodysplastic syndrome (MDS)/refractory anemia with excess blasts, type 2 (RAEB2). The patient underwent two hematopoietic stem cell transplantations (HSCT) and achieved a stable remission after the second transplantation. Bone marrow samples were available from different phases of the disease as follows: 1) diagnosis before any therapy (15% blasts), 2) first relapse after allogenic HSCT (6 % blasts), 3) before unresponsive treatment with idarubisin, AraC, and thioguanine (20% blasts), and 4) remission after second allogenic HSCT (3% blasts). The sample taken at diagnosis was subjected to whole-genome sequencing and all post-treatment samples to RNA-sequencing. Cultured skin fibroblasts from the patient were available as a matched normal reference.

Hospital-based series of hematological malignancies

Three hundred sixty-seven index cases with hematological malignancies, including 166 with acute myeloid leukemia (AML), 52 with acute lymphoblastic leukemia (ALL), 19 with myelodysplastic syndrome (MDS), 107 with multiple myeloma, and 23 with myeloproliferative neoplasms (MPN) were available [5]. Fibroblastoid samples and leukemic bone marrow samples from the patients were investigated to generate 367 germline exomes and 432 somatic exomes.

DNA and RNA were extracted from fresh or fresh-frozen samples as described [1], and from formalin-fixed paraffin-embedded (FFPE) specimens by the protocol of Isola et al. [6] Traces of genomic DNA were eliminated from RNA samples by a variety of methods, including spin column purification and DNaseI treatment. This study was approved by the institutional review boards of the Helsinki University Hospital, Helsinki, Finland (approval nos. 466-46-2001, 206/13/03/03/2016 and 303/13/03/01/2011). The National Supervisory Authority for Welfare and Health (Valvira/Dnro 10741/06.01.03.01/2015) approved the collection of archival specimens.

**Exome sequencing (ES)**

Institute for Molecular Medicine Finland, FIMM (Helsinki, Finland) carried out ES on DNA from index persons and 1 – 2 additional family members when available. ES was performed on Illumina HiSeq 2000 platform with Roche NimbleGen SeqCap EZ Exome Library V2. For primary and secondary data analysis, a variant calling pipeline (VCP 3.2) developed by FIMM [7] was used. Paired-end reads were aligned to the GRCh37/Hg19 human genome build by using the Burrows-Wheeler Alignment (BWA). ES statistics for the three FCCTX families with *DHX40*, *TDRD9*, or *TDRD5* variants is available in Supplementary Table 1.

ES for hematological samples was conducted at the Institute for Molecular Medicine Finland, FIMM (Helsinki, Finland) using Nimblegen SeqCap EZ v2 or MedExome (Roche, Basel, Switzerland), Agilent SureSelect v5 Exome or XT Clinical Research Exome (Agilent, Santa Clara, CA, USA capture kits as described previously [5, 8]. The sample series and ES studies thereof have been previously reported in Wartiovaara-Kautto et al. [8], Douglas et al. [9], Hakkarainen et al. [10], and Lahtinen et al. [11].

**Genome sequencing (GS)**

GS was performed on DNAs from individuals IV.3 (blood) and V.1 (bone marrow and skin fibroblasts) from family 32. Additionally, individual III.3 (blood) who later turned out to be a non-carrier for the *DHX40* c.710_713delTCAG variant was studied by GS. ThruPLEX® DNA-seq Kit (Rubicon Genomics) was used according to Nieminen et al. [12] on Illumina HiSeq 2500 equipment. An in-house variant calling pipeline VCP3.4 [7] was used for primary and secondary data analysis. Paired-end reads were aligned to the GRCh37/Hg19 human genome build as described above for ES. GS statistics is available in Supplementary Table 1.

**ES and GS data analysis to detect germline variants**

Primary and secondary data analysis was carried out at FIMM as described above. For tertiary analysis, VarSeq® (Golden Helix, for FCCTX samples) and BasePlayer software (<https://baseplayer.fi/>, for hematological samples) were used for filtering out common variants. Only high quality (Phred-scale likelihood <70), rare (MAF <0.001), and nonsynonymous variants (frameshift, stop gained/lost, missense, disrupting donor/acceptor site variants within ± 2 bp of the exon end) were selected for further analysis. Autosomal dominant inheritance was assumed. Missense variants were predicted using six different *in silico* protein prediction softwares (SIFT, PolyPhen-2, MutationAssessor, MutationTaster, FATHMM, and FATHMM Coding). Variants with a maximum of one prediction as tolerated were taken into account. Next, the variants were checked against the SISu database containing variants for 10490 Finnish samples ([www.sisuproject.fi](http://www.sisuproject.fi)) and against Ensembl (grch37.ensembl.org), gnomADv2.1.1 (gnomad.broadinstitute.org) and GEEVS (geevs.crg.eu) databases, and variants with MAF > 0.001 were removed. Additionally, gene function and expression data were obtained from the GeneCards database (<http://www.genecards.org/>).

Additionally, the next-generation sequencing data were evaluated for possible deletions encompassing one or several exons by the R package ExomeDepth [13] Copy number changes were also addressed by using a genome-wide SNV array as described below.

**Genome wide single-nucleotide variant (SNV)-array for copy number variants (CNV)**

DNA samples from the index patients of FCCTX families were genotyped following the array manufacturer’s instructions on Illumina Infinium Omni5-4 v.1.1 array that covers >4.3 million variants with the median distance of 0.36 kb from each other. The arrays were run using Illumina iScan –scanner (Illumina, San Diego, CA). The genotypes were called with Illumina GenomeStudio 2.0 –software. The following QC steps were performed during GenomeStudio analysis: Approximately 15 000 SNVs were manually inspected based on several threshold values covering e.g. call rates, signal intensities, number of heterozygotes, separation of genotype clusters and number of clusters. Additional QC steps (e.g., SNV success rates and gender check) were performed utilizing Plink v1.9 software package.

CNVs were called using Illumina CNVPartition 3.1.6 add-on program. CNV regions were visually inspected using Genome Viewer utility of the GenomeStudio 2.0 program. PennCNV software tool was used to call CNV regions shared among the samples based on B allele frequencies and LogR values obtained from GenomeStudio 2.0.

The genotype success rates for the samples after discarding totally failed SNVs were 99.7 – 99.9 %. All SNVs with success rates under 90% were removed from analysis. 1851376 SNVs showed no variation in this dataset. Genotypes from a total of 12996 variants out of 4284426 were discarded during QC, but B allele frequencies and LogR ratios for these samples were retained. Map positions of the SNVs used in genotyping / analysis were based on GRCh37.p13 / hg19, and the alleles were named according to Illumina’s top/bottom strand orientation.

**Sanger sequencing**

Confirmation of all relevant sequence changes detected by ES or GS and co-segregation investigations were by Sanger sequencing on genomic DNA. Optimal primer designs utilized Primer3 program (<http://bioinfo.ut.ee/primer3-0.4.0/>) and sequences are available from the authors upon request.

**RNA-sequencing**

RNA-sequencing was performed on bone marrow samples from individual V.1 from F32. FIMM carried out the sequencing as previously described [5] on Illumina HiSeq 2000 equipment. An in-house pipeline for RNA-sequencing, VCP 2.5, developed by FIMM was used [14].

**RNA-sequencing of cell lines for expression and splicing analyses**

For each cell line (HEK293, CCD841CoN, and K562), triplicate samples of siRNA-treated (treated with *DHX40*-specific siRNA) and “untreated” cells (treated with siRNA buffer instead of siRNA to control for DharmaFECT toxicity), together with negative (treated with non-target siRNA) and positive controls (treated with *GAPDH*-siRNA) were extracted using NucleoSpin® RNA extraction kit (Macherey-Nagel, Düren, Germany) and subjected to Whole transcriptome RNA-sequencing at Functional Genomics Unit (FuGU, Helsinki, Finland) using NovaSeq S4 platform. Library for RNAseq was prepared using Illumina Stranded Total RNA Prep with Ribo-Zero Plus kit.

Differential transcript expression (DTE) analyses were carried out on Salmon-quantified [15] RNA-seq data using the Fishpond R package [16]. Treatment-specific variances did not statistically differ from each other. Downstream analyses were conducted on untreated versus siRNA treated data.

We applied the ASGAL (Alternative Splicing Graph Aligner) tool [17] to Salmon-aligned and quantified RNA-seq data to detect novel alternative splicing events with respect to gene annotation.

**Somatic variant analysis from ES, GS, or RNA-sequencing data**

To detect somatic non-synonymous variants, GS and RNA-seq data on bone marrow samples from patient V.1 from F32 were evaluated against germline data derived from GS analysis on DNA from the patient’s skin fibroblasts. Analysis was conducted by Baseplayer setting minimum quality score 0, minimum genotype quality 0, minimum coverage 4, and minimum allelic fraction 1% as requirements. To exclude common germline variants possibly originating from allogenic bone marrow donors (relevant for RNA-seq samples for which no germline data for the bone marrow donors were available), only variants with population frequencies less than 0.001 were considered.

VarScan2 variant detection algorithm version 2.3.2 [18] was applied to neoplastic tissue-normal tissue pairs to identify non-synonymous somatic variants from WES or WGS data. Annotation of the variants was done using SnpEff version 4.0 with the Ensembl v68 annotation database (https://www.ensembl.org). Variants with a somatic *p*-value less than 0.01 were selected.

Somatic variants presented in Figure 2E were called from publicly available datasets of colorectal, myeloid, and lymphatic neoplasia reported in cBioPortal [19-29]. Missense-type variants were filtered based on their pathogenicity prediction by the same software as described above for germline variants.

**Single Nucleotide Primer Extension (SNuPE) for allele-specific expression (ASE) of candidate genes in blood RNA**

The basic principle of SNuPE is described in Renkonen et al. [1] The germline variant was used as a target to design gDNA and cDNA-specific PCR reactions. PCR products served as templates for primer extensions with three dNTPs and one ddNTP, resulting in extension products that separated the wild-type and mutant allele by allele length. To investigate expression consequences of the *DHX40* c.710_713delTCAG variant, gDNA was amplified with forward primer 5’-aatagtttggctgttaagtgtac-3’ (intron 4) and reverse primer 5’-cacccaattagtaagaatcatag-3’ (intron 5) and extension primer was FAM-5’-atatacctggaaggctttatc-3’ (exon 5). To generate cDNA, total cellular RNA was extracted from blood using PAXgene^TM^ Blood RNA Kit v2 (PreAnalytiX, Hombrechtikon, Switzerland), and from lymphoblasts or fibroblasts using RNAeasy minikit (Qiagen, Chatsworth, California, USA). RNA samples were reverse transcribed with VILO Superscript cDNA Synthesis kit (Thermo Fisher Scientific, Waltham, Massachusetts, USA) according to manufacturer’s instructions. For PCR, 400 ng of cDNA was used in each reaction. For cDNA amplification, forward primer 5’-ttcgttttgatgattgcagttc-3’ (exon 3) and reverse primer 5’-cacaaagacatcttcgtagtac-3’ (exon 15) were used, and the extension primer was the same as for gDNA reaction above. In both gDNA and cDNA reactions, the stopping nucleotide was ddT. Extension products of 32 bp and 25 bp were expected for the mutant (Mut) and wild-type (WT) allele, respectively. ASE ratio was determined as the ratio of the peak area of the mutant (Mut) to the wild-type (WT) allele in cDNA relative to gDNA and calculated as R = (Mut/WT)_cDNA_/(Mut/WT)_gDNA_. Values R<0.6 or R>1.67 were considered to indicate ASE [12].

**Loss of heterozygosity (LOH) analysis**

Paired normal and tumor tissues were used for LOH analysis. LOH at the site of the *DHX40* c.710_713delTCAG variant was examined by fragment analysis of amplification products generated with forward primer FAM-5’-tggaattagccaagctctct-3’ and reverse primer 5’-gccaagtaataccgcttgaa-3’. The products were run on ABI 3730 XL. The ratios of the peak areas of mutant (Mut) to wild-type (WT) alleles in tumor DNA (T) relative to normal DNA (N) were calculated using the following equation: LOH ratio = (Mut/WT)_T_/(Mut/WT)_N_. LOH ratios ≤ 0.60 and ≥ 1.67 were considered to indicate LOH (≥ 40 % decrease of one allele compared to the other) [30].

**Bisulfite sequencing of *DHX40***

To determine the methylation status of the promoter region of *DHX40* [31], bisulfite primers were designed to cover the 713 bp CpG island (GRCh37; 17:57 642 684-57 643 397) in *DHX40* identified by the EMBOSS CpGplot tool. Methylation-unbiased primers were designed and bisulfite conversion was carried out as described by Niskakoski et al. [32]. The CpG island was divided into four PCR reactions: DHX40_BS1 5’-TGTTAAGTGTGTGAAGGTAGAATAGTTTG-3’ (forward), 5’-ACTCCACCCCCCAAAAATTTCC-3’ (reverse), DHX40_BS2 5’-TAAGAGGGGGTTTTYGGGTTTT-3 (forward), 5’-CTCTTCCTAAAAATCTCTTAAC-3’ (reverse), DHX40_BS3 5’-TTTTATTYGGGGTTAGGTTTATGTTT-3’ (forward), 5’-TACTTTCATCCCCCCACCAAAACCCCCA-3’ (reverse), DHX40_BS4 5’-GGAGGAGGGTGAGYGGTT-3’ (forward) and 5’-AAAAAATCTATAAACCTATTTAAAAACAAAATAC-3’ (reverse).

**Methylation-Specific Multiplex Ligation-dependent Probe Amplification (MS-MLPA) analysis of *DHX40***

Guided by bisulfite sequencing results, three MS-MLPA probe pairs were designed following instructions by MRC-Holland (<http://mrc-holland.com>) for methylation analyses of *DHX40* in patient (FFPE) specimens. The probe pairs were as follows: DHX40_1 LPO-GGGTTCCCTAAGGGTTGGACATCTTGAGTCCATCTCAGAGACCACTGTGGCGTTGAAAAGAGG, DHX40_1 RPO-TGTCGTCGCGACCTTCGGCGCCGGAGAGGCTTCATGGTTCATGGTTCATCTAGATTGGATCTTGCTGGCAC, DHX40_2 LPO-GGGTTCCCTAAGGGTTGGACATCTTGAGTCCACACGTCATCGAGCAGCTCCCCCTCCCCTTGC, DHX40_2 LPO-TACAAGTCGCACGCGCGGAAGTAAACACTTCATGGTTCTCTAGATTGGATCTTGCTGGCAC, DHX40_3 LPO-GGGTTCCCTAAGGGTTGGACATCTTGAGTCCATCTTACGTCATCAGGGCGCGTCCTCGTCTTTC, DHX40_3 RPO-CCCTCCCATCTCCTCAGATCGGTGGACGTGCTTCTAGATTGGATCTTGCTGGCAC.

**Cloning experiment to determine allele-specificity of *DHX40* methylation**

The SNV 17:57642846 delG (rs10713316) located in the *DHX40* promoter was used as a marker of the wild-type allele, since it was present in individual V.1 from F32 but absent in her affected mother IV.3 and grandfather III.1 who were all heterozygous for the pathogenic c.710_713delTCAG variant. DNA extracted from the bone marrow sample at diagnosis from individual V.1 was bisulfite-converted using EZ DNA Methylation-Direct Kit (Zymo Research, CA, United States) and a fragment of 362 bp encompassing the SNV was amplified with methylation-unbiased primers DHX40_BS1 forward and DHX40_BS2 reverse. The PCR product was cloned using the TOPO® TA Cloning® Kit for Subcloning (Thermo Fisher, MA, United States) as described in Olkinuora et al. [33] DNA extracted from the clones was sequenced and the ratio of methylated to unmethylated alleles with the presence vs. absence of the SNV determined.

**Immunohistochemical (IHC) analysis of DHX40 expression**

FFPE-derived tissue sections were stained with DHX40 antibody NBP1-91834 (rabbit polyclonal antibody against C-terminal amino acids 635 – 760) from Novus Biologicals (Littleton, Colorado USA), in 1:100 dilution. Dako Envision+ System, DAB Peroxidase was applied according to manufacturer’s instructions for visualization.

**Western blot analysis of DHX40 expression in patient samples and in HEK293 cell line transfected with expression constructs**

Total protein was extracted from lymphoblastoid cells of III.1 and lymphoblastoid and fibroblast cells of control individuals. Protein separation was performed by electrophoresis on Novex (Carlsbad, CA) NuPage 4%-12% Bis-Tris Gels (1.0 mm) and blotted on nitrocellulose membranes (BioRad). Primary antibody against DHX40, C-terminal (anti-rabbit Anti-DHX40, NBP1-91834, 1:500 dilution, Novus Biologicals, CO) or N-terminal (rabbit polyclonal Anti-DHX40 antibody, 7.4 μg/μl; TA331741, OriGene Technologies) and secondary antibodies (rabbit polyclonal antibody (1:1000 dilution, FL-335; Santa Cruz Biotechnology) were hybridized and then visualized with Amersham (Buckinghamshire, UK) ECL Prime Western Blotting Detection reagents and scanned with a CCR camera. The housekeeping protein glyceraldehyde-3-phosphate dehydrogenase (ab128915, Abcam, Cambridge, UK) was used as a loading control.

Plasmid constructs (pcDNA3.1+N-eGFP) containing either wild-type *DHX40* or c.710_713delTCAG and an N-terminal eGFP tag were ordered from GenScript. HEK293 cells were transfected using TurboFectin 8.0 transfection reagent (OriGene, Technologies, MD, USA) according to manufacturer’s instructions. Briefly, HEK293 cells were plated at a density of 0.5 x 10^6^ in 6-well plate in antibiotic-free complete growth medium and grown overnight at 37°C in 5% CO_2_. The following morning, 15 µl of TurboFectin 8.0 was added to 250 µl of Opti-MEM with 5 µg of plasmid DNA, and incubated 15 minutes at RT. The mixture was then added dropwise to the cells and incubated 24 or 48 hours at 37°C in 5% CO_2_ after which the cells were passaged into complete media with 600 µg/ml G418 for a week until non-transfected cells perished. Total protein was extracted from transfected and control HEK293 cells using LAEMMLI buffer and protein separation was performed as described above. Anti-Enhanced Green Fluorescent Protein (eGFP; ABIN678503, Antibodies-Online GmbH, Aachen, Germany) was used for detection of plasmid products. GAPDH was used as a loading control.

**Small interfering RNA (siRNA) treatment of cell lines**

CCD841CoN (ATCC-CRL-1790, normal colon epithelium from fetus), and K562 cells (ATCC-CCL-243, bone marrow from chronic myeloid leukemia) were purchased from ATCC and HEK293 (catalogue number 300192, immortalized human embryonic kidney cells) from Cell Lines Services (CLS). Authentication by genotyping at FIMM verified their identity.

Custom pool of siRNA oligos for *DHX40* was designed to target the non-pseudogene homologous regions in exons 4 and 5 of *DHX40* and ordered from Horizon Discovery (Cambridge, United Kingdom) along with a pool of ON-TARGETplus Non-targeting siRNA and a pool of ON-TARGETplus siRNAs targeting the housekeeping gene *GAPDH.* According to the manufacturer’s instructions, transfection was optimized for each cell line to keep the viability > 80 % in triplicate. Knockdown of *DHX40* and *GAPDH* was confirmed by qRT-PCR. Briefly, 5, 6 or, 5 µl of each *DHX40* siRNA oligo (5µmol), and 12.5, 5 or, 5 µl of *GAPDH* and Non-targeting siRNA (5µmol) were diluted in 10% of the total growth volume Opti-MEM for HEK293 (10 ml), CCD841CoN (4 ml), and K562 (4 ml) cells, respectively. In a separate tube, 25, 8 or 8 µl of DharmaFECT was diluted in 10% of total volume of growth medium. After incubating the mixtures in RT for 5 min, the siRNA mixture was added to the transfection reagent mixture, and incubated for a further 20 min in RT. The old growth medium was removed from cells, and the siRNA-transfection reagent mixture was added in appropriate amount of antibiotic-free complete growth medium. Cells were then incubated at 37°C in 5% CO_2_ for 24 hours, after which the cells were collected for sample extraction. 1 x Dharmacon^TM^ siRNA Buffer (Horizon Discovery, Cambridge, United Kingdom) was used as a negative control.

**Statistical analyses**

Statistical analyses were carried out using either IBM® SPSS® software (IBM SPSS Statistics 27, Armonk, NY: IBM Corp) or appropriate R packages. Two-tailed *p*-values were calculated and, when appropriate, corrected for multiple testing. FDR corrected *p* values < 0.05 were considered statistically significant, unless stated otherwise.

For analysis of novel splicing events by ASGAL, Independent-samples Median Test was applied for the number and genomic size of reads supporting a splicing event using IBM SPSS software.

**Code availability**

Any code used to generate results can be requested from the corresponding author at alisa.olkinuora@helsinki.fi.

1. Renkonen E, Zhang Y, Lohi H, Salovaara R, Abdel-Rahman WM, Nilbert M*, et al.* Altered expression of MLH1, MSH2, and MSH6 in predisposition to hereditary nonpolyposis colorectal cancer. *J Clin Oncol.* 2003;21**:**3629-3637.

2. Vasen HF, Mecklin JP, Khan PM, Lynch HT. The International Collaborative Group on Hereditary Non-Polyposis Colorectal Cancer (ICG-HNPCC). *Diseases of the colon and rectum.* 1991;34**:**424-425.

3. Vasen HFA, Watson P, Mecklin JP, Lynch HT. New clinical criteria for hereditary nonpolyposis colorectal cancer (HNPCC, Lynch syndrome) proposed by the International Collaborative Group on HNPCC. *Gastroenterology.* 1999;116**:**1453-1456.

4. Umar A, Boland CR, Terdiman JP, Syngal S, Chapelle Adl, Ruschoff J*, et al.* Revised Bethesda Guidelines for Hereditary Nonpolyposis Colorectal Cancer (Lynch Syndrome) and Microsatellite Instability. *Journal of the National Cancer Institute.* 2004;96**:**261-268.

5. Malani D, Kumar A, Brück O, Kontro M, Yadav B, Hellesøy M*, et al.* Implementing a Functional Precision Medicine Tumor Board for Acute Myeloid Leukemia. *Cancer Discov.* 2022;12**:**388-401.

6. Isola J, DeVries S, Chu L, Ghazvini S, Waldman F. Analysis of changes in DNA sequence copy number by comparative genomic hybridization in archival paraffin-embedded tumor samples. *Am J Pathol.* 1994;145**:**1301-1308.

7. Sulonen AM, Ellonen P, Almusa H, Lepistö M, Eldfors S, Hannula S*, et al.* Comparison of solution-based exome capture methods for next generation sequencing. *Genome Biol.* 2011;12**:**R94.

8. Wartiovaara-Kautto U, Hirvonen EAM, Pitkänen E, Heckman C, Saarela J, Kettunen K*, et al.* Germline alterations in a consecutive series of acute myeloid leukemia. *Leukemia.* 2018;32**:**2282-2285.

9. Douglas SPM, Lahtinen AK, Koski JR, Leimi L, Keränen MAI, Koskenvuo M*, et al.* Enrichment of cancer-predisposing germline variants in adult and pediatric patients with acute lymphoblastic leukemia. *Sci Rep.* 2022;12**:**10670.

10. Hakkarainen M, Koski JR, Heckman CA, Anttila P, Silvennoinen R, Lievonen J*, et al.* A germline exome analysis reveals harmful POT1 variants in multiple myeloma patients and families. *EJHaem.* 2022;3**:**1352-1357.

11. Lahtinen AK, Koski J, Ritari J, Hyvärinen K, Koskela S, Partanen J*, et al.* Clinically relevant germline variants in allogeneic hematopoietic stem cell transplant recipients. *Bone Marrow Transplant.* 2023;58**:**39-45.

12. Nieminen TT, Pavicic W, Porkka N, Kankainen M, Järvinen HJ, Lepistö A*, et al.* Pseudoexons provide a mechanism for allele-specific expression of APC in familial adenomatous polyposis. *Oncotarget.* 2016;7**:**70685-70698.

13. Plagnol V, Curtis J, Epstein M, Mok KY, Stebbings E, Grigoriadou S*, et al.* A robust model for read count data in exome sequencing experiments and implications for copy number variant calling. *Bioinformatics.* 2012;28**:**2747-2754.

14. Kumar A, Kankainen M, Parsons A, Kallioniemi O, Mattila P, Heckman CA. The impact of RNA sequence library construction protocols on transcriptomic profiling of leukemia. *BMC Genomics.* 2017;18**:**629.

15. Patro R, Duggal G, Love MI, Irizarry RA, Kingsford C. Salmon provides fast and bias-aware quantification of transcript expression. *Nat Methods.* 2017;14**:**417-419.

16. Zhu A, Srivastava A, Ibrahim JG, Patro R, Love MI. Nonparametric expression analysis using inferential replicate counts. *Nucleic Acids Res.* 2019;47**:**e105.

17. Denti L, Rizzi R, Beretta S, Vedova GD, Previtali M, Bonizzoni P. ASGAL: aligning RNA-Seq data to a splicing graph to detect novel alternative splicing events. *BMC Bioinformatics.* 2018;19**:**444.

18. Koboldt DC, Larson DE, Wilson RK. Using VarScan 2 for Germline Variant Calling and Somatic Mutation Detection. *Curr Protoc Bioinformatics.* 2013;44**:**15 14 11-17.

19. Giannakis M, Mu XJ, Shukla SA, Qian ZR, Cohen O, Nishihara R*, et al.* Genomic Correlates of Immune-Cell Infiltrates in Colorectal Carcinoma. *Cell Rep.* 2016;15**:**857-865.

20. Gao J, Aksoy BA, Dogrusoz U, Dresdner G, Gross B, Sumer SO*, et al.* Integrative analysis of complex cancer genomics and clinical profiles using the cBioPortal. *Sci Signal.* 2013;6**:**pl1.

21. Cerami E, Gao J, Dogrusoz U, Gross BE, Sumer SO, Aksoy BA*, et al.* The cBio cancer genomics portal: an open platform for exploring multidimensional cancer genomics data. *Cancer Discov.* 2012;2**:**401-404.

22. Seshagiri S, Stawiski EW, Durinck S, Modrusan Z, Storm EE, Conboy CB*, et al.* Recurrent R-spondin fusions in colon cancer. *Nature.* 2012;488**:**660-664.

23. Ellrott K, Bailey MH, Saksena G, Covington KR, Kandoth C, Stewart C*, et al.* Scalable Open Science Approach for Mutation Calling of Tumor Exomes Using Multiple Genomic Pipelines. *Cell Syst.* 2018;6**:**271-281.e277.

24. Roelands J, Kuppen PJK, Ahmed EI, Mall R, Masoodi T, Singh P*, et al.* An integrated tumor, immune and microbiome atlas of colon cancer. *Nat Med.* 2023;29**:**1273-1286.

25. Vasaikar S, Huang C, Wang X, Petyuk VA, Savage SR, Wen B*, et al.* Proteogenomic Analysis of Human Colon Cancer Reveals New Therapeutic Opportunities. *Cell.* 2019;177**:**1035-1049.e1019.

26. Landau DA, Tausch E, Taylor-Weiner AN, Stewart C, Reiter JG, Bahlo J*, et al.* Mutations driving CLL and their evolution in progression and relapse. *Nature.* 2015;526**:**525-530.

27. Chapuy B, Stewart C, Dunford AJ, Kim J, Kamburov A, Redd RA*, et al.* Molecular subtypes of diffuse large B cell lymphoma are associated with distinct pathogenic mechanisms and outcomes. *Nat Med.* 2018;24**:**679-690.

28. Papaemmanuil E, Gerstung M, Bullinger L, Gaidzik VI, Paschka P, Roberts ND*, et al.* Genomic Classification and Prognosis in Acute Myeloid Leukemia. *N Engl J Med.* 2016;374**:**2209-2221.

29. Puente XS, Beà S, Valdés-Mas R, Villamor N, Gutiérrez-Abril J, Martín-Subero JI*, et al.* Non-coding recurrent mutations in chronic lymphocytic leukaemia. *Nature.* 2015;526**:**519-524.

30. Ollikainen M, Abdel-Rahman WM, Moisio A-L, Lindroos A, Kariola R, Järvelä I*, et al.* Molecular Analysis of Familial Endometrial Carcinoma: A Manifestation of Hereditary Nonpolyposis Colorectal Cancer or a Separate Syndrome? *Journal of Clinical Oncology.* 2005;23**:**4609-4616.

31. Magzoub MM, Prunello M, Brennan K, Gevaert O. The impact of DNA methylation on the cancer proteome. *PLoS Comput Biol.* 2019;15**:**e1007245.

32. Niskakoski A, Kaur S, Staff S, Renkonen-Sinisalo L, Lassus H, Järvinen HJ*, et al.* Epigenetic analysis of sporadic and Lynch-associated ovarian cancers reveals histology-specific patterns of DNA methylation. *Epigenetics.* 2014;9**:**1577-1587.

33. Olkinuora AP, Mayordomo AC, Kauppinen AK, Cerliani MB, Coraglio M, Collia Á K*, et al.* Mono- and biallelic germline variants of DNA glycosylase genes in colon adenomatous polyposis families from two continents. *Front Oncol.* 2022;12**:**870863.
