# Supplementary material for: Identification of DHX40 as a candidate susceptibility gene for colorectal and hematological neoplasia
Source: Leukemia. 2023 Sep 11;37(11):2301–5. doi: 10.1038/s41375-023-02021-9 (PMC10624609; doi:10.1038/s41375-023-02021-9)
Supplement: Supplementary file 2 — Legends for Supplementary Figures [file 41375_2023_2021_MOESM2_ESM.docx]

**Legends for Supplementary Figures**

**Supplementary Figure 1.**

Consequences of the *DHX40* c.710_713delTCAG variant on mRNA and protein expression, as well as the wild-type allele status in tumor tissue. (A) Single nucleotide primer extension analysis utilizing the germline variant to evaluate allele-specific mRNA expression in variant carriers IV.3 (lymphoblasts) and V.1 (fibroblasts) from family 32 and a healthy control (blood). Peaks specific to the wild-type (WT) and mutant (Mut) allele are shown. Expression from the mutant allele (arrow) relative to the wild-type allele was reduced, as indicated by the Mut to WT peak area ratios of 0.19 (IV.3) and 0.36 (V.1) in cDNA when normalized with the corresponding peak area ratios in genomic DNA (gDNA). A similar degree of expressional reduction for the mutant allele was evident by RNA-seq (data not shown). (B) Fragment analysis to assess loss of the wild-type allele in tumor tissue from individuals IV.3 (colon adenoma) and III.1 (colon carcinoma) from family 32; blood DNA from a healthy control is also shown. The ratio of the mutant to wild-type allele-specific peaks in tumor DNA, normalized with the respective peak area ratio in normal DNA, was close to 1 in both patients, indicating retention of the wild-type allele. (C) Immunohistochemical analysis to determine DHX40 protein expression in tumor tissues from individuals IV.3 (colon adenoma) and IV.5 (breast carcinoma) from family 32, and in normal colonic mucosa from an unrelated control. Solid arrows denote areas of tumor cells, and open arrows areas of non-tumorous cells. Normal mucosa and lymphoid cells from the control individual showed strong positive staining, whereas stroma was negative. Adenomatous tissue from IV.3. showed mosaic staining (the section contained no stromal tissue). Lobular breast carcinoma *in situ* cells from IV.5 were DHX40-positive and stroma was negative.

**Supplementary Figure 2.**

Western blot analysis of DHX40 protein expression. Figure 2A shows results from clinical specimens, including lymphoblastoid cells from individual III.1 with *DHX40* c.710_713delTCAG variant, and lymphoblastoid (LyBl) and fibroblastoid (FiBl) cells from control individuals. Hybridization was with N-terminal (left) or C-terminal (right) antibody against DHX40. GAPDH was used as a loading control. Solid arrowheads indicate DHX40 isoforms present in patient and control samples, with some sample- or tissue-type-specific variation between relative quantities of the isoforms. Figure 2B displays results from the HEK293 cell line transfected with eGFP-tagged wild-type (WT) or mutant (delTCAG) *DHX40* expression constructs. Hybridization was with anti-eGFP. Expression from the wild-type construct resulted in a protein of expected size of ~130 kDa (solid arrowhead), whereas expression from the mutant construct gave rise to a truncated product of ~60 kDa (open arrowhead).

**Supplementary Figure 3.**

Bar graph of functionally annotated, differentially expressed transcripts unique to *DHX40*-siRNA-treated cells. Cell lines HEK293, CCD841, and K562 shared differential expression for these transcripts (n = 71), and results are shown separately for each cell line. Primary categorization of the transcripts was according to function. The top to bottom order of transcripts within each group follows the magnitude of expressional change shown on the horizontal axis and expressed as mean Log2FC change vs. untreated cells. Underexpressed transcripts (mean Log2FC below zero) are on the left and overexpressed transcripts on the right (mean Log2FC above zero). A majority of the transcripts were underexpressed.
