# Supplementary figures and images for: Identification of DHX40 as a candidate susceptibility gene for colorectal and hematological neoplasia
Source: Leukemia. 2023 Sep 11;37(11):2301–5. doi: 10.1038/s41375-023-02021-9 (PMC10624609; doi:10.1038/s41375-023-02021-9)

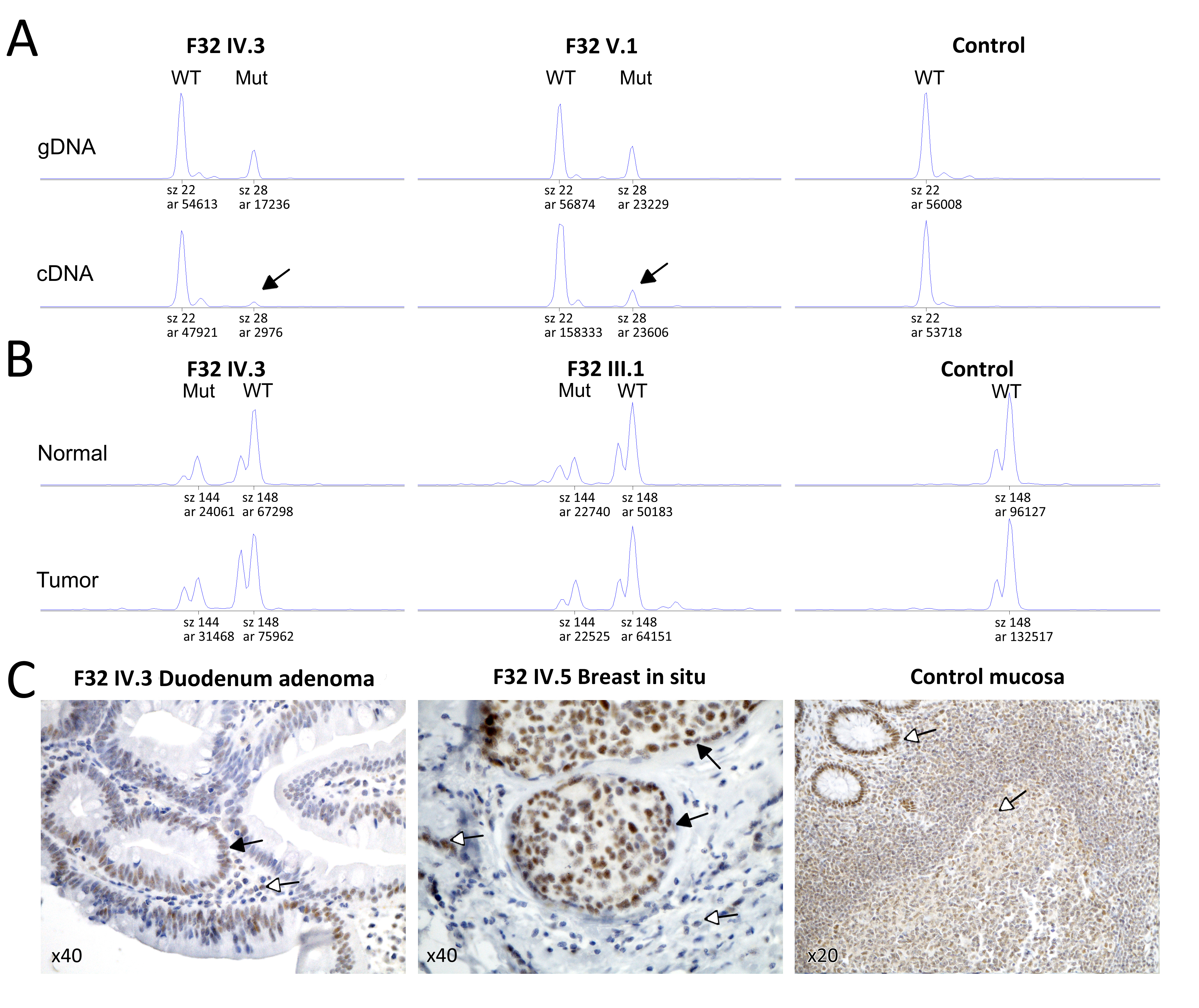

Supplement: Supplementary file 3 — Supplementary Figure 1 [file 41375_2023_2021_MOESM3_ESM.tif]

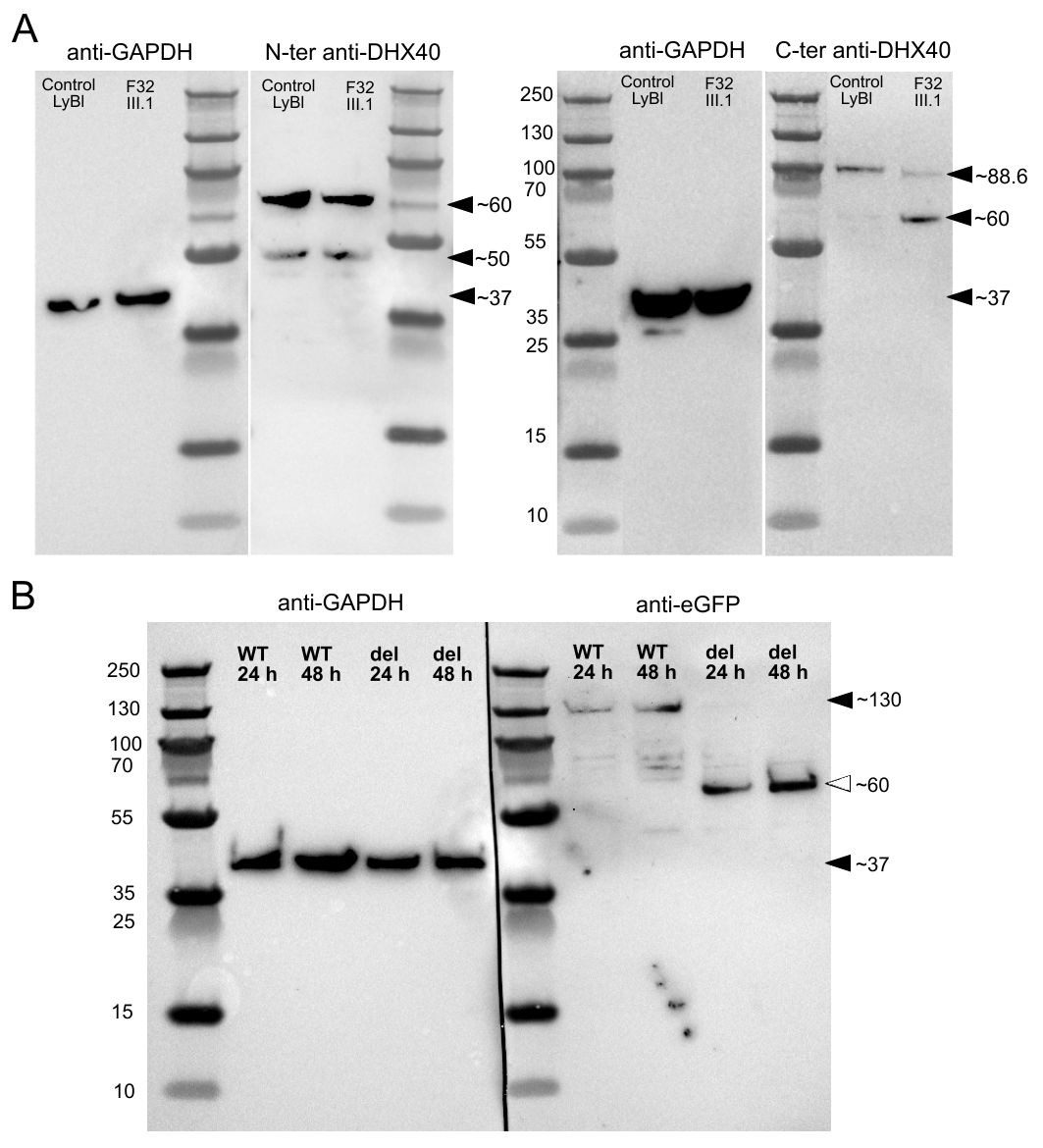

Supplement: Supplementary file 4 — Supplementary Figure 2 [file 41375_2023_2021_MOESM4_ESM.tif]

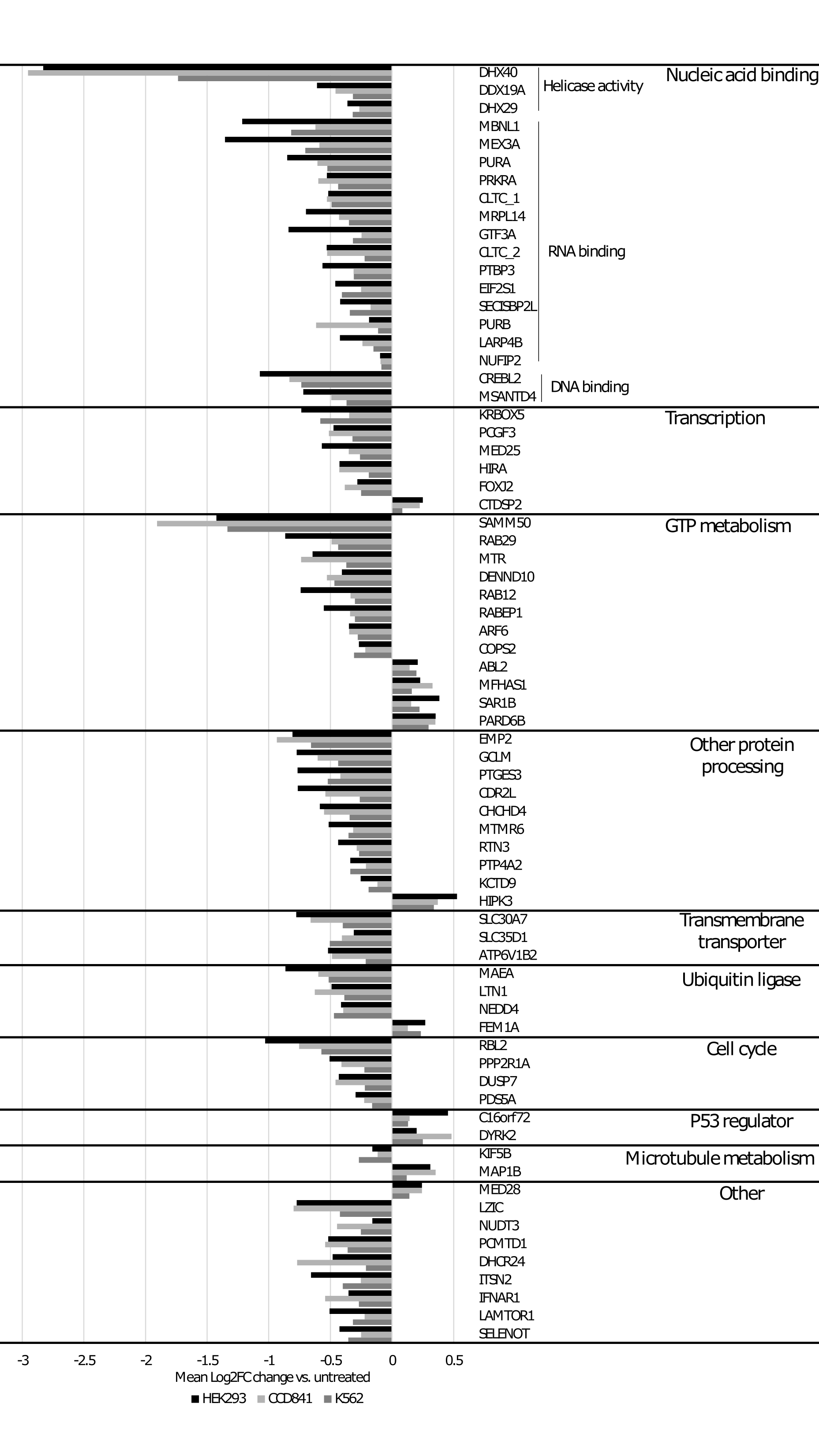

Supplement: Supplementary file 5 — Supplementary Figure 3 [file 41375_2023_2021_MOESM5_ESM.jpg]
